# Clinical Characteristics and Controllable Risk Factors of Osteoporosis in Elderly Men with Diabetes Mellitus

**DOI:** 10.1111/os.12957

**Published:** 2021-04-05

**Authors:** Chunling Li, Shufang Wang, Mengru Du, Yihan Wei, Sheng Jiang

**Affiliations:** ^1^ Department of Endocrinology The Second Affiliated Hospital of Tianjin Traditional Chinese Medicine University Tianjin China; ^2^ Department of Endocrinology Cao Xian People's Hospital of Shandong Province Heze China; ^3^ State Key Laboratory of Pathogenesis, Prevention and Treatment of High Incidence Diseases in Central Asia; Department of Endocrinology The First Affiliated Hospital of Xinjiang Medical University Urumqi China

**Keywords:** Clinical characteristics, Elderly men, Hazardous factors, Osteoporosis type‐2 diabetes

## Abstract

**Objective:**

To analyze the clinical characteristics and controllable risk factors of osteoporosis in elderly men with type‐2 diabetes mellitus (T2DM).

**Methods:**

A total of 250 elderly OP patients with T2DM were included in the present study. Patients with one or more common chronic diseases (including hypertension, coronary heart disease, heart failure, chronic bronchitis, chronic nephrosis, and cirrhosis), and a course of more than 3 years were defined as complicated with chronic diseases. Blood glucose, cholesterol, triglyceride, low‐density lipoprotein, high‐density lipoprotein, calcium, phosphorus, glycosylated hemoglobin, urea nitrogen, creatinine, fasting insulin, liver function, and 25‐hydroxy vitamin D3 levels were measured. Bone mineral density was also measured.

**Results:**

A total of 16 patients (6.4%) had severe osteoporosis. Furthermore, 66 patients (26.4%) had blood glucose control that reached the standard, while 176 patients (70.4%) used more than two anti‐diabetic drugs. The serum testosterone level was lower than the median in 87 patients (34.8%) and in 56 smokers (22.4%). Furthermore, 138 patients (55.2%) were overweight and obese, six patients (2.4%) were underweight, 197 patients (78.8%) had chronic diseases, 88 patients (35.2%) were sticking to exercise, and 117 patients (46.8%) had less exercise. In addition, 92 patients (36.8%) were treated with osteotrophy‐protective agents, and 24 patients (9.6%) received anti‐osteoporosis therapy. Smoking, poor glycemic control, low testosterone levels, less exercise, and complications with chronic diseases were the most relevant controllable risk factors.

**Conclusion:**

For elderly male osteoporosis patients with type‐2 diabetes, smoking cessation, blood sugar control up to the standard, regular exercise, active prevention and treatment of complications, and appropriate testosterone supplementation are necessary for preventing and curing osteoporosis.

## Graphical abstract

Technology roadmap

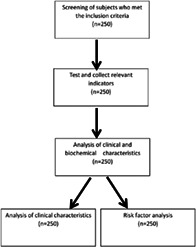

## Introduction

Osteoporosis is a kind of systemic bone disease characterized by decreased bone mass and damaged microstructure of the bone, which results in increased osteopsathyrosis and vulnerability to fracture. This disease can be divided into two categories: primary osteoporosis and secondary osteoporosis^1^. Primary osteoporosis (OP) is an age‐related chronic disease with many atypical clinical symptoms. Therefore, this presently has a low awareness rate and treatment rate. In 2016, the prevalence of OP in elderly people over 60 years old was 36% in China, in which 23% were men. This seriously threatens the health status of the elderly^2^. At present, China is a country with the largest elderly population in the world. This means that the absolute number of patients with T2DM and OP is also one of the largest^3^. An early epidemiological survey revealed that the prevalence of osteoporosis in Chinese people over 50 years of age is 20.7% in women and 14.4% in men, and the prevalence of osteoporosis in people over 60 years old is significantly increased^4^. The greatest risk of osteoporosis is also the most serious consequence of osteoporotic fractures, which are also known as fragility fractures. The common sites include the vertebral bodies, hips, distal forearm, proximal humerus, and pelvis. Among these, vertebral body fractures are most common^5^. In 2016, the prevalence of osteoporosis in elderly people over 60 years old was 36% in China, among which 23% were men and 49% were women. This indicates that osteoporosis has become an important public health problem in China^6^. OP is an age‐related metabolic disease, and its incidence increases with age^7^. Osteoporosis may occur in old people, regardless of gender. However, since the peak bone mass of men is higher than that of women, the age of bone loss is later than that of women, and the decrease in androgen level is “progressive” rather than “free‐fall.” Furthermore, the amount and speed of bone loss in older men is lower than those in older women, the degree of osteoporosis in older men is lighter than that in women, and osteoporosis in older men is also of the low‐conversion type^8^. Therefore, the overall age of osteoporosis in elderly men is later than that in women, and the severity is lower than that in women^9^, which has its own characteristics. Type‐2 diabetes mellitus (T2DM) is one of the risk factors for OP, and one of the most common concomitant diseases in elderly patients^10^. There is a mutual aggravation and mutual causality in elderly OP patients who have T2DM at the same time, which is one of its own characteristics, and should be identified in prevention and treatment strategies.

T2DM is a chronic metabolic disease and an age‐related disease. It is also one of the risk factors for OP^11^. Hence, elderly OP patients complicated with T2DM are at a stage of aggregation due to multiple risk factors of OP. Furthermore, this group of people have their own characteristics. Our goal is to analyze the clinical characteristics and controllable risk factors of osteoporosis in elderly men with T2DM.

## Methods

### Researching Object

#### Inclusion Criteria

The inclusion criteria for this study are as follows: (i) people who were diagnosed with T2DM in accordance with the World Health Organization (WHO 1999) definition^12^; (ii) elderly patients, defined as patients with an age of ≥60 years old; (iii) people who were diagnosed with OP in accordance with the diagnostic criteria recommended in the Guidelines for the Diagnosis and Treatment of Osteoporosis in the Elderly in China (2018)^13^, ^14^.

#### Exclusion Criteria

The exclusion criteria for this study are as follows: (i) patients with acute diabetic complications; (ii) patients with severe hepatic and renal insufficiency, abnormal endocrine and metabolic disorders, digestive tract diseases and malignant tumors; (iii) patients who received adrenal glucocorticoids and androgens, and drugs that could affect bone metabolism; (v) patients with mobility difficulties, infirmity of age and communication barriers, and those unable to cooperate with the completion of all examinations; (iv) patients who were unwilling to cooperate with the completion of the questionnaire.

#### Demographic Data

A total of 250 elderly OP patients with T2DM were included in the present study. These patients were treated in the endocrine ward from the first affifiliated hospital of Xinjiang medical university January 2018 to November 2019, had a mean age of 72.3 ± 8.4 years old, and a course of T2DM of 8.7 ± 3.2 years. The following data were measured to analyze the clinical characteristics and controllable risk factors of osteoporosis in elderly men with T2DM: (i) the clinical characteristics and (ii) controllable risk factors of osteoporosis in elderly men with type‐2 diabetes mellitus. Smoking was defined as more than three cigarettes per day, which lasted for over 3 months. Exercise for less than 150 min per week was considered as less, while exercise not less than three times a week, not less than 30 min each time was considered as regular exercise. Patients with one or more common chronic diseases and a course of more than 3 years were defined as complicated with chronic diseases. Common chronic diseases mainly included hypertension, coronary heart disease, heart failure, chronic bronchitis, chronic nephrosis, and cirrhosis. The diagnostic criteria were all referred to the relevant guidelines recommended by the Chinese Medical Association. The diagnosis of patients being overweight and/or obese was based on the criteria recommended in the Guidelines for the Prevention and Treatment of Type‐2 Diabetes formulated by Diabetes Branch of the Chinese Medical Association.

#### Ethical Statement

The present study protocol was approved by the hospital medical science ethics committee, and patients provided signed informed consent.

### Baseline Data of the Subjects

The height, weight, blood pressure, and body mass index (BMI) were measured by specially trained medical staff using unified standards under fasting in the morning. A questionnaire was made to collect the smoking history, past history, and other related information. The information was collected from uniform questionnaires.

#### Blood Biochemical Tests

Venous blood was drawn from all the subjects in the morning after fasting for 12 h. The blood samples were measured for blood glucose, cholesterol, triglyceride, low‐density lipoprotein, high‐density lipoprotein, calcium, phosphorus, glycosylated hemoglobin, urea nitrogen, creatinine, fasting insulin, liver function, and 25‐hydroxy vitamin D3 levels using the automatic biochemical instrument in the hospital clinical laboratory on the same day.

#### Bone Mineral Density Measurement

The bone densitometry was carried out at the hospital's bone mineral density (BMD) laboratory. This was performed by specialists using dual‐energy X‐ray absorptiometry, in order to measure the lumbar and femoral neck BMD in g/cm^2^, with a coefficient of variation <1%. The diagnosis report for the test results was uniformly issued by the operation technician.

### Statistical Analysis

The statistical analysis was conducted using the SPSS 21.0 statistical software. Normally distributed data were measured using the normality test, and expressed as mean ± standard deviation (SD). The risk factors were analyzed by logistic regression analysis, and *P* < 0.05 was considered statistically significant.

## Results

### Basic Information of Patients

The baseline data stratified by age are presented in Table 1. Among the 250 included elderly OP patients with T2DM, 16 patients suffered from severe OP, accounting for 6.4%. Furthermore, 66 patients had blood glucose control up to the standard (glycosylated hemoglobin <7.0%), accounting for 26.4%, 176 patients took more than two anti‐diabetic drugs, accounting for 70.4%, the serum testosterone level was lower than the median in 87 patients, accounting for 34.8%, and 56 patients who were smokers accounted for 22.4%. In addition, 138 patients were overweight and obese, accounting for 55.2%, while six patients were underweight, accounting for 2.4%. Moreover, 197 patients (78.8%) had chronic diseases, 88 patients maintained their exercise routine, accounting for 35.2%, 117 patients performed less exercise, accounting for 46.8%, 92 patients were treated with osteotrophy‐protective agents, accounting for 36.8%, and 24 patients received anti‐osteoporosis treatment, accounting for 9.6%.

**TABLE 1:** Baseline data for 250 patients (Mean ± SD)

Risk factors	60 ≤ Age < 70 (years)	70 ≤ Age < 80 (years)	≥80 (years)
	82 people	103 people	65 people
Course of T2DM (year)	6.5 ± 2.2	8.4 ± 4.2	9.4 ± 5.3
Number of Hba1c < 7.0%	20	18	30
Hypoglycemic agent >2 kinds	49	61	65
Number of Low testosterone	18	29	39
Smokers	28	19	9
BMI≥25 Kg/m^2^	49	65	24
BMI < 19 Kg/m^2^	2	3	1
Chronic diseases	37	57	103
Less exercise	16	32	48
Regular exercise	39	25	24
Osteotrophy‐protective agents	24	29	39
Anti‐osteoporosis treatment	4	8	12

Bone health supplement: Calcium and vitamin D; BMI, body mass index.; Hba1c, glycosylated hemoglobin.

### Multi‐factor Non‐conditional Logistic Analysis in Elderly Osteoporosis Patients with Type‐2 Diabetes

Combined with the clinical characteristics of this group, the main common risk factors were age, drinking history, diabetes course, testosterone level, being overweight or obese, being underweight, smoking, complications with chronic diseases, and lack of exercise. The multi‐factor non‐conditional logistic analysis revealed that the controllable risk factors for OP consisted of smoking (OR = 1.395. *P* < 0.01), poor glycemic control (OR = 2.21. *P* < 0.01), low testosterone levels (OR = 1.56. *P* < 0.01), less exercise (OR = 1.42. *P* < 0.01), and complications with chronic diseases (OR = 1.44. *P* < 0.01) (Table 2).

**TABLE 2:** Multi‐factor non‐conditional logistic analysis in elderly osteoporosis patients with type‐2 diabetes

Variable	B	SE	Wald	OR	*P‐*value
Smoking	−0.111	0.017	46.43	1.395	<0.01
Low testosterone levels	0.58	0.169	11.80	1.56	<0.01
Hba1c > 7.0%	1.184	0.154	58.63	2.21	<0.01
Associated chronic disease	0.617	0.142	15.83	1.44	<0.01
Regular exercise	0.368	0.157	5.40	1.42	<0.01

## Discussion

The present study revealed that among the 250 elderly OP male patients with T2DM, 26 patients had severe osteoporosis, which accounted for 10.4%. This suggests that the proportion of severe osteoporosis is not high in this group of people, considering factors such as the slow decline of sex hormones in older men relative to women. There were 66 patients with blood glucose control up to the standard, accounting for 26.4%, and 146 patients used more than two kinds of anti‐diabetic drugs, accounting for 58.4%. This suggests that the poor control of blood sugar with a lower target rate is one of the factors for the progression of OP. Blood sugar control should be strengthened to increase the target rate. The treatment was more complex in nearly 60% of patients with diabetes, which is also the reason for the poor control of blood sugar. This indicates that special attention should be given to multi‐factor interventions in this population, in order to achieve comprehensive standards. Among the 250 elderly men, 87 patients had lower testosterone than the median, accounting for 34.8%. This indicates that this physiological phenomenon is also one of the reasons for the occurrence and development of OP. There were 108 overweight and obese patients, accounting for 43.2%. This suggests that weight management needs to be strengthened, and that more attention should be given to metabolic abnormalities caused by being overweight or obese. Therefore, this population has the phenomena of aggregation of OP risk factors, low awareness rate, and low treatment rate. Poor glycemic control and various complications of diabetes may have a negative effect on bone mineral density. The abnormal energy metabolism, vitamin D, electrolytes, and other organ functions of diabetes mellitus and its acute and chronic complications can lead to osteoporosis. Some anti‐diabetic drugs may also lead to different levels of osteoporosis. Therefore, T2DM is one of the risk factors for OP, and one of the most common concomitant diseases in elderly patients^15^.

OP risk factors include genetic factors and environmental factors. The identification and intervention of risk factors are the main measures to prevent and treat OP. The risk factors of OP can be divided into uncontrollable factors and controllable factors. Among these, the uncontrollable factors are mainly race, aging, female menopause, family history of fragility fracture, and existing complications^16^. The main controllable factors consist of unhealthy lifestyles, including less physical activities, smoking, excessive drinking, excessive drinking of caffeinated drinks, nutritional imbalance, excessive or insufficient protein intake, calcium and/or vitamin D deficiency, high sodium diet, low body mass, unreasonable use of drugs, etc. The present study revealed that although there were many risk factors in elderly OP patients with T2DM, among these controllable risk factors, smoking, substandard blood glucose control, low testosterone, and less exercise were the most important risk factors that need attention. A limitations of this study is its relatively small sample size.

### Conclusion

With the continuous progression of aging, the prevention and treatment of chronic diseases in the elderly population has gained increasing concern, and the number of elderly male OP patients with T2DM continues to increase. Analyzing the characteristics of such a population and minimizing the controllable risk factors would be beneficial to its targeted prevention and treatment, and the improvement of the health level and quality of life of these patients. Smoking cessation, blood sugar controlled up to the standard, regular exercise, and appropriate testosterone supplementation are necessary in order to prevent and cure osteoporosis in this group of people.

## Funding

This work was supported by the fund project in the State Key Laboratory of Pathogenesis, Prevention and Treatment of High Incidence Diseases in Central Asia (No.SKL‐HIDCA‐2019‐15).

## Disclosure

We have no competing interests.
